# QEVO^®^-Assisted Anatomical Inspection of Adjacent Perforators in Microsurgical Clipping—Technical Note

**DOI:** 10.3390/brainsci15030300

**Published:** 2025-03-12

**Authors:** Adi Ahmetspahic, Eldin Burazerovic, Hana Rizvanovic, Ema Selimovic, Eleonora Kujaca, Mirza Pojskic, Alberto Feletti, Kenan Arnautovic

**Affiliations:** 1 Department of Neurosurgery, Clinical Center of University of Sarajevo, 71000 Sarajevo, Bosnia and Herzegovina; adi.ahmetspahic@ssst.edu.ba (A.A.); eldindon@yahoo.com (E.B.); 2 Department of Medicine, University Sarajevo School of Science and Technology, 71000 Sarajevo, Bosnia and Herzegovina; hana.rizvanovic@stu.ssst.edu.ba (H.R.); ema.selimovic@stu.ssst.edu.ba (E.S.); eleonora.kujaca@stu.ssst.edu.ba (E.K.); 3 Department of Neurosurgery, Philipps University of Marburg, 35043 Marburg, Germany; 4 Department of Neurosciences, Biomedicine, and Movement Sciences, Institute of Neurosurgery, University of Verona, 37126 Verona, Italy; alberto.feletti@univr.it; 5 Department of Neurosurgery, The University of Tennessee Health Science Center, Memphis, TN 38138, USA; kenanarnaut@yahoo.com

**Keywords:** intracranial aneurysm, endoscope, microsurgery, aneurysm clipping

## Abstract

Introduction: Aneurysms of brain vessels are life-threatening conditions with various adverse outcomes, some stemming from microsurgical intervention, particularly when major vessel perforators are inadequately protected. The use of endoscopes enhances the approach to aneurysms by providing closer visualization (180–360 degrees) of the local anatomy, potentially reducing accidental damage. To improve visualization and efficiency, a microscope-integrated 45-degree angled microinspection endoscopic tool (QEVO^®^, Carl Zeiss, Oberkochen^TM^) has been developed and employed in various neurosurgical procedures. Methods: Between 2021 and 2025, 27 brain aneurysms were treated with QEVO^®^ assistance at the Department of Neurosurgery, Clinical Center of the University of Sarajevo. The choice of the videos corresponds to the best image quality in videos and on the microscopic determination of adjacent vessel perforators, which were not adequately seen purely by the surgical microscope in specific cases. Exclusion criteria included cases without a need for QEVO^®^ assistance in perforator visualization, severe brain edema, intraoperative aneurysm rupture, posterior circulation, or low video quality. Results: Case 1 demonstrates an anterior choroidal artery (AchA) aneurysm; Case 2 presents an anterior communicating artery (AcommA) aneurysm; and Case 3 features contralateral middle cerebral artery (MCA) microsurgical clipping with QEVO^®^ assistance. Conclusions: The QEVO^®^ tool significantly improves the visualization of aneurysm–perforator relationships, increasing the likelihood of preserving perforators during standard microsurgical clipping. This innovative approach may reduce surgical complications and enhance patient outcomes, highlighting the tool’s potential as an adjunct in aneurysm microsurgery.

## 1. Introduction

Cerebral aneurysms encompass life-threatening conditions in neurosurgery, often leading to various adverse outcomes. Occasionally, the origin of unfavorable results may be attributed to the complications arising during microsurgical intervention. To maximize the success of the aneurysm occlusion, the neck of the affected artery should be completely clipped, while the main, branching, and perforating arteries should be conserved. With the help of an endoscope, this may be achieved more efficiently [1]. The benefits of an endoscope are reflected in enhancing the approach to the aneurysm itself, yielding better visualization of the surrounding anatomy and perforating vessels, as well as a reduction in accidental brain or blood vessel damage. However, the utilization of endoscopes may bring additional costs; it is time consuming, and the number of endoscopic neurosurgeons is not high [2,3]. Nevertheless, modern microsurgery has increased the use of endoscopic assistance in many centers over the previous years. The first report of the utilization of an endoscope during microsurgery dates back to the 1970s for the treatment of hypophysial lesions. In the late 1990s, the concept of endoscopic microsurgical assistance was introduced to intracranial lesions, notably in the posterior fossa. Finally, by the end of 1998, minimally invasive surgery had been popularized by Perneczky, and it has been in use increasingly since then [4]. The need for novel approaches in close visualization has led to the development of the QEVO^®^ microinspection device (Carl Zeiss AG^TM^, Oberkochen, Germany), a 12 cm, 45-degree angled endoscopic tool with a 100-degree field of view. Its purpose is to expand visualization without interfering with the regular operative efficiency during microsurgical procedures. The Zeiss KINEVO 900^®^ (Carl Zeiss AG^TM^, Oberkochen, Germany) operating microscope and the QEVO^®^ equipment are fully integrated, allowing surgeons to switch between endoscopic and microscopic views easily. The QEVO^®^ device is able to be plugged into the KINEVO 900^®^ microscope console directly while the produced image is displayed on a high-resolution monitor. The benefits of QEVO^®^ include easy integration, high functionality of suction-instrument shape, and the lack of the need to additionally use separate endoscopic apparatus [5].

QEVO^®^-assisted microsurgery has been in use since 2017 [6]. Considering its particulate polyvalency, its role in the broader diapason of neurosurgical operations is still in question. Up until now, we have lacked research papers that describe methods of QEVO^®^ assistance precisely, especially in aneurysm surgery.

## 2. Materials and Methods

This study was conducted at the Department of Neurosurgery of the Clinical Center of the University of Sarajevo (CCUS) between 2021 and 2025. Around 70 aneurysms are treated in our department yearly, with a significant microsurgical vs. endovascular ratio. The decision regarding the utilization of QEVO^®^ is primarily based on the availability of a specialized team capable of performing either procedure. The 27 aneurysms included in this study were cases in which the first author was exclusively present during the surgery, with additional selection criteria defined by specific exclusion criteria. The endoscopic-assisted technique, consistent with the QEVO^®^ application, adopted by the author himself during a cerebrovascular fellowship at Fujita Health University, Nagoya, Japan, was transferred to our department and has been used in aneurysm cases since 2021. Both ruptured and unruptured aneurysms were included in this study. Further selection criteria were based on intraoperative vessels’ anatomical clarity and the possibility of management of the aneurysm, without the risk of an untoward course of the surgery. Not enough space for the instrument, clear microscopic anatomical aneurysm–perforator relation, severe brain edema, intracerebral hematoma, posterior circulation as well as intraoperative rupture of the aneurysm were exclusion criteria. The radiological outcome was assessed in a three-dimensional computer tomography angiography (3D CTA) through Workstation IDS7, version 25.1.15.4485, Sectra AB^®^ and included intraoperative videos.

## 3. Results

**Figure 1:**
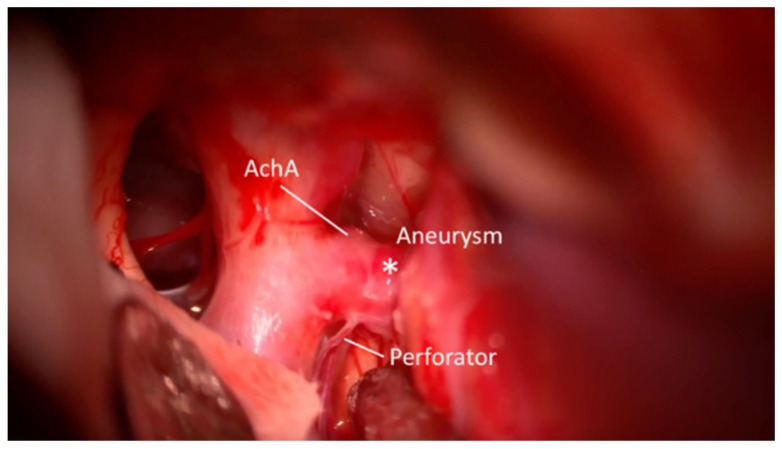
Microscopic view; Initial part of the ICA Ach-A aneurysm pointed laterally and a single perforator distally are visible. *—Aneurysm.

**Figure 2:**
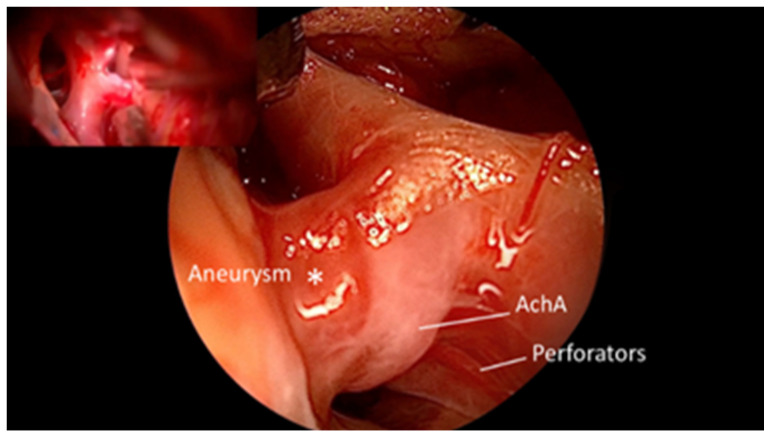
QEVO^®^-assisted view on the anterior side i.e., proximally; Perforator from the right ICA-PCom is hidden below the ICA-AchA, which is not visible microscopically (preclipping state). *—Aneurysm.

**Figure 3:**
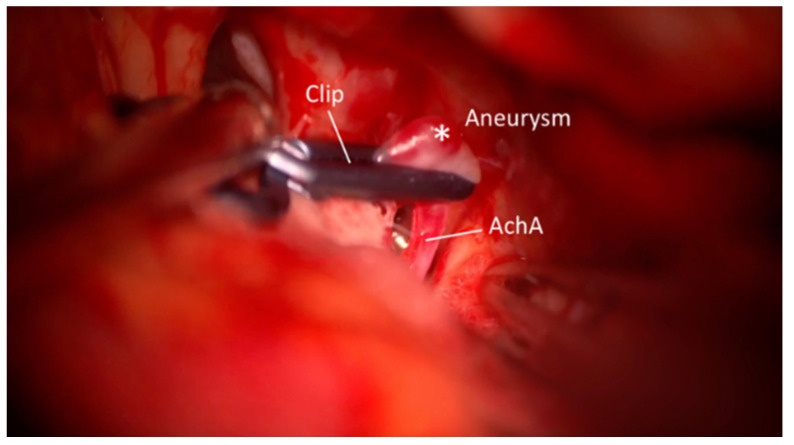
Microscopic view shows clipped aneurysm without possibility of inspecting the integrity of the AchA; perforators are not adequately visible. *—Aneurysm.

**Figure 4:**
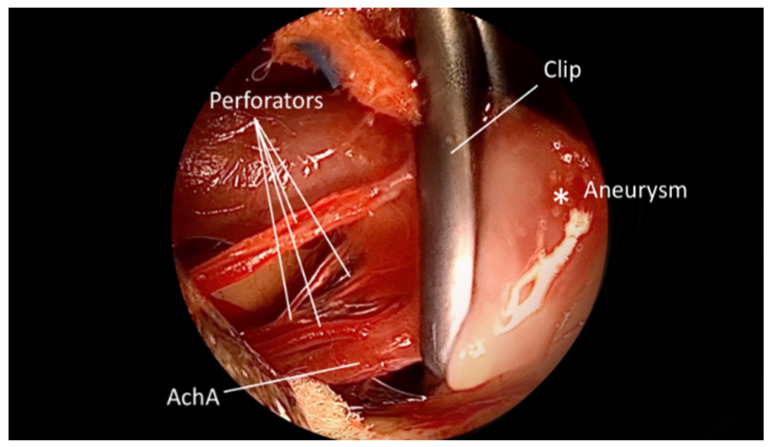
QEVO^®^ view dorsal side i.e., distally from the AchA; Clip excluded aneurysm, while perforators as well as the right AchA are free. *—Aneurysm.

**Figure 5:**
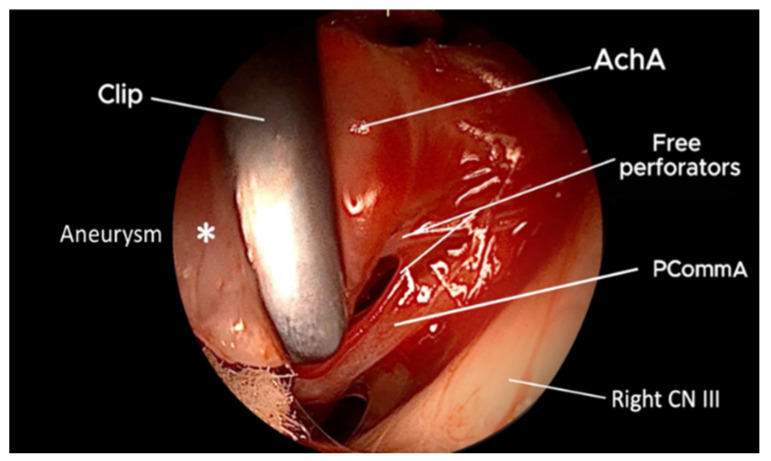
QEVO^®^ view ventral side i.e., proximally from the clip; the origin of the AchA is intact, while the PCommA and the perforators are free. *—Aneurysm.

**Figure 6:**
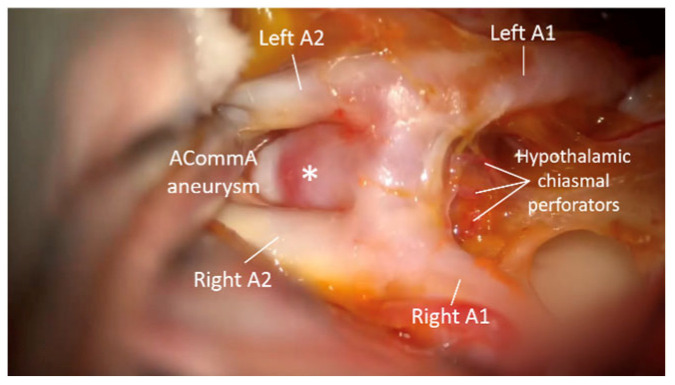
Microscopic view; ACoA aneurysm pointing upwards and posteriorly is seen, as well as small hypothalamic perforators below the ACoA complex (Precliping state). *—Aneurysm.

**Figure 7:**
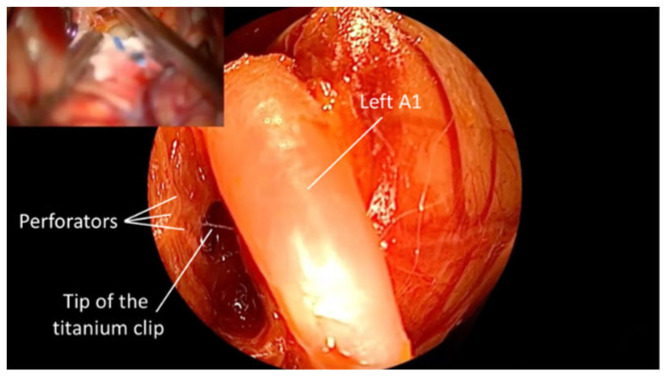
Microscopic image in the left upper corner demonstrates forceps of the clip on the previously noted aneurysm, without possibility of visualization of the tip of the clip. The relation of perforators to the tip of the clip is not visible. QEVO^®^ view from bellow the AcoA complex demostrates the tip of the clip in relation to free perforators.

**Figure 8:**
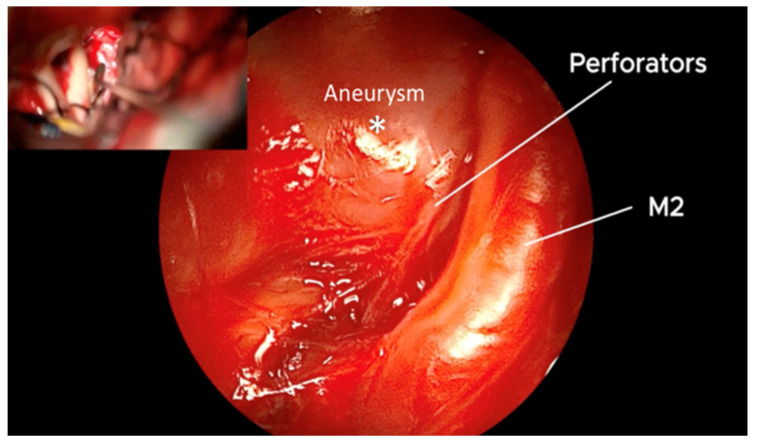
The microscopic image in the left upper corner does not provide visualization of the perforators noted below, with QEVO^®^-assisted inspection (large image). Therefore, there is a possibility of damaging the visualized perforators (preclipping state). *—Aneurysm.

**Figure 9:**
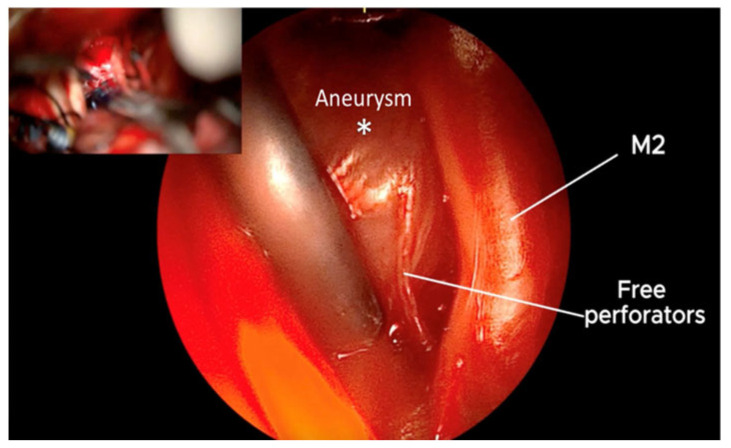
QEVO^®^-assisted inspection of clip positioning reveals free, undamaged perforators, and the clipped MCA aneurysm. This is in contrast to the microscopic image above, where only the clip and the body of the aneurysm are seen. *—Aneurysm.

Three different aneurysm locations are presented as follows: Case one represents a right anterior choroidal artery (ICA—AchA) unruptured aneurysm (Figure 1, Figure 2, Figure 3, Figure 4 and Figure 5); 

Case two represents a ruptured anterior communicating artery (ACommA) aneurysm (Figure 6 and Figure 7); 

Case three represents a contralateral unruptured middle cerebral artery aneurysm (MCA), after clipping of the ipsilateral ruptured left fusiform posterior communicating artery (ICA—PCommA) aneurysm, treated in an identical setting through unilateral craniotomy (Figure 8 and Figure 9).

There were no intraoperative complications related to the device in any of the procedures, and none of the perforators or magistral blood vessels were excluded from the circulation in endoscopic view. No temporary clipping was used, and no clip readjustment was needed in any of the cases. Further, no residual aneurysm neck was noted in 3D postoperative CTA. Operative videos are presented in a supplemental file format for each case.

## 4. Discussion

To prevent ischemic consequences, the goal of aneurysm surgery is effective occlusion while maintaining every single branching or passing blood vessel. Therefore, each aneurysm site’s vascular anatomy could be adjusted from an endoscopic standpoint [2,7].

The endoscope’s benefits are its simultaneous ability to visualize some blind regions and to produce a clear image of nearby and distant structures [8]. According to Kato et al., endoscopic assistance includes observations of areas that are not visible with a microscope such as the perforating arteries passing along the aneurysm and the remaining neck, which was not visible with the initial clip in place [9]. Also, the implementation of endoscope assistance can potentially decrease postoperative complications and minimize the necessity for brain retraction [5,10]. In terms of image quality, we found that QEVO^®^ assistance offered a broad-angle view without shortcomings. Schebesch et al. published a larger series with a detailed description of QEVO^®^ implementation in aneurysm clipping [2], which was not our goal. We focused on the local inspection of perforators before and after the clipping. What is novel in our work is the contralateral simultaneous use of QEVO^®^ and micro-clipping of the MCA, which, to our knowledge, has not been previously published. ICA—AchA aneurysms could be heavily involved with perforators, posing the greatest risk of perforator injury. Its delicate nature makes it particularly susceptible to damage, which can lead to complications; for example, Bohnstedt et al. found that using a temporary clip and the frequency of its application were linked to ischemic events. Therefore, temporary clipping should only be employed when necessary and with caution, aiming to aid in the final aneurysm dissection [11,12]. Also, as the vascular territory of the anterior choroidal artery (AchA) varies reciprocally with the supply from the posterior communicating artery (PCommA) and middle cerebral artery (MCA), the thorough endoscopic identification of possible anatomical variations is important for the preservation of perforators. Therefore, we present QEVO^®^ assistance, in which the aneurysm clipping was performed with preservation of the surrounding perforators and main trunk of the AChA without the need for a temporary occlusion or clip readjustment (Video S1).

ACommA aneurysm clipping is linked to some limitations, particularly in ruptured cases. Those limitations involve the inadequate observation of the relation between the aneurysm dome and the hypothalamic-chiasmal perforators, especially in superiorly and backward-oriented aneurysms [13,14]. We found QEVO^®^ assistance overcomes those limitations by providing an inspection of the most hidden postero-basal part of the ACommA complex, in which the tip of the clip and its relation toward perforators may be inspected (Video S2).

In the context of mirror MCA aneurysms, there are instances in which, with carefully selected criteria, the clipping and addressing of contralateral aneurysms can be achieved using a unilateral approach [15]. Its clipping might seem unsafe due to the restricted visualization, long dissection distances, and poor maneuverability in a narrow space. However, Rodríguez-Hernández et al. suggest that clipping of the contralateral MCA aneurysm is feasible and generally easier than it seems [16]. Those aneurysms that are directed upward or downward are easier to clip because they align with the surgeon’s line of sight. Conversely, aneurysms that are directed sideways along the axis of the MCA are the most challenging. We believe surgeons could feel discomfort in contralateral aneurysm exposure, which can result in refraining from the final clip occlusion of the aneurysm, especially if the aneurysm is large or has a wide neck. It may happen that the microscope alone does not provide a sufficiently clear visualization according to the visual obstruction by the contralateral optic nerve or frontobasal cortex in cases of edema. Nevertheless, when cerebral circumstances are unfavorable, the contralateral approach is advised to be avoided [3,17]. In our earlier report on the clinical characteristics of aneurysms, we showed the highest incidence of MCA and more than 10% of aneurysm multiplicity in our patients [18]. Although contralateral MCA aneurysm clipping is rare, we treat four to five similar cases annually. When it comes to the stage of clipping, the question remains what may be found at “*the dark side of the aneurysm*”, i.e., contrary to the side of the microscopic view. In these particular situations, we found that QEVO^®^ assistance comes to its full expression to overcome the limitations of the microscopic view. In our case, the team of two vascular neurosurgeons worked simultaneously; one had experience in endoscopic handling and guided another surgeon to properly and safely place the final clip. By this modification, we established that simultaneous micro-endoscopic control over the aneurysm’s surrounding structures might be extremely helpful in preserving tiny perforators, preventing them from being accidentally involved in the tip of the clip (Case 3). To the best of our knowledge, our case offers a unique presentation with no previous information on endoscopic-guided aneurysm clipping that could be found in the literature.

### Limitations vs. Advantages

According to our research, QEVO^®^ was sufficient to access the anterior circulation even in deeper locations of the anterior circulation, such as contralateral aneurysm of the MCA via a unilateral approach (Video S3). However, the ideal case for a device application is an unruptured ipsilateral aneurysm with previously performed microvascular dissection, which ensures adequate space for the manipulation of any endoscope, such as QEVO^®^ [3]. Conversely, its application can be extremely difficult in cases of unmanageable brain edema or in the presence of an intracerebral hematoma, i.e., limited manipulative space, which constituted one of the exclusion criteria in our study. In this regard, another limiting factor can be a bloody surgical field, as well as a fogged lens on the microinspection instrument produced by debris. In the presented cases (1–3), we were required to frequently clean the instrument tip and reposition it away from potential sources of minor venous or cortical bleeding. The exploration of relevant anatomy for the given pathology at the moment is of the utmost importance, along with maintaining a clear field. To achieve this, continuous irrigation and suction of the operative field should be practiced.

Further, the instrument’s rigidity restricts certain maneuvers and sometimes necessitates a fixed position relative to the microsurgical field, similar to the rigid endoscope. Although this study was not designed as a comparative, we did not observe any trouble in the prior technical use of a rigid endoscope vs. the QEVO^®^ endoscope for pure microsurgical assistance. The advantage of using the QEVO^®^ device was evident in eliminating the need for full endoscopic equipment for assistance in selected cases, allowing the rigid endoscope to be utilized for another purpose. However, in the intention to access below the arteries or behind the aneurysm, where most of the perforators were hidden, the inflexibility of the instrument poses another risk of injury to critical structures, requiring careful handling. In our cases, we consistently maintained the instrument at a safe distance from the aneurysm wall or major blood vessels to minimize any risk of damage. While the rigidity of the device limits exploration, it offers key advantages, including excellent illumination, which, according to our experience, provides sufficient information about the local anatomical specificities of perforating arteries. The crucial advantages of the QEVO^®^ microinspection tool are evident during two critical stages: inspection of the relationship of the aneurysm neck and local perforators before clipping and inspection of the perforators alone after clipping. The adequate microsurgical dissection of surrounding structures and the aneurysm neck is the key to ensuring sufficient space for QEVO^®^ visualization. This is of particular importance for perforators on the dorsal side of the aneurysm, which are inaccessible with the microscopic view, as seen in Videos S1-3. Looking ahead, flexible scopes have been reported in which endoscopic assistance was utilized even for tumor resection [19]. The potential adoption of a flexible microinspection tool suggests a promising direction for future advancements in microsurgical-assisted clipping.

## 5. Conclusions

QEVO^®^-assisted clipping represents a modest approach in the microsurgical management of aneurysms. Although microsurgical clipping is considered the standard method in the management of cerebral aneurysms, the benefits of QEVO^®^ cannot be overlooked. This study offered new advances for deep-seated aneurysms in terms of simultaneous micro-endoscopic guided clipping techniques. While the utilization of QEVO^®^ cannot be recommended as a standard technique yet, in selected cases and experienced hands, it offers superior endoscopic visualization of locally exposed anatomy and microscopic hidden structures.

## Data Availability

Portions of the data present in this study were presented at the Bosnian–Herzegovinian American Academy of arts and sciences (BHAAAS) in Sarajevo, as an oral presentation titled “QEVO-Assisted microsurgery—A Technical Note”. Parts of it were also presented at the Asian Congress of Neurological Surgeons (ACNS) webinar in 2023. At the 9th SNSS Annual Meeting and the 10th SNSS Congress in 2023, the data were presented under the title “Pathway of the Young Neurosurgeon in the field of Vascular and Skull Base Surgery”. Further data are available upon request from the corresponding author.

## Supplementary Materials

The following supporting information can be downloaded at https://www.mdpi.com/article/10.3390/brainsci15030300/s1, Video S1: QEVO®-assisted microsurgical clipping of the ICA AChA aneurysm; Video S2: QEVO®-assisted microsurgical clipping of the ACommA aneurysm; Video S3: QEVO®-assisted microsurgical clipping of the contralateral MCA aneurysm.

## Abbreviations

The following abbreviations are used in this manuscript:

AchA	Anterior choroidal artery
ACommA	Anterior communicating artery
ICA	Internal carotid artery
MCA	Middle cerebral artery
PCommA	Posterior communicating artery
